# Network analysis and functional estimation of the microbiome reveal the effects of cashew nut shell liquid feeding on methanogen behaviour in the rumen

**DOI:** 10.1111/1751-7915.13702

**Published:** 2020-11-09

**Authors:** Koki Maeda, Van Thu Nguyen, Tomoyuki Suzuki, Keita Yamada, Kushi Kudo, Chie Hikita, Van Phong Le, Minh Chon Nguyen, Naohiro Yoshida

**Affiliations:** ^1^ Crop, Livestock & Environment Division Japan International Research Center for Agricultural Sciences (JIRCAS) 1‐1 Ohwashi Tsukuba Ibaraki 305‐8686 Japan; ^2^ Faculty of Agriculture Can Tho University Campus II, 3/2 St Ninh Kieu Can Tho Vietnam; ^3^ Central Region Agricultural Research Center National Agriculture and Food Research Organization (NARO) 768 Senbonmatsu Nasu‐shiobara Tochigi 329‐2793 Japan; ^4^ Department of Environmental Chemistry and Engineering Tokyo Institute of Technology 4259 Nagatsuta Midori‐ku Yokohama 226‐8502 Japan; ^5^ Faculty of Human Development and Environment Kobe University 3‐11 Tsurukabuto Nada‐ku Kobe 657‐8501 Japan; ^6^ Idemitsu Kosan, Co. Ltd. 2‐1 Midorigahara Tsukuba Ibaraki 300‐2646 Japan; ^7^ Earth‐Life Science Institute Tokyo Institute of Technology 2‐12‐1 Ookayama, Meguro‐ku Tokyo 152‐8550 Japan

## Abstract

The effects of cashew nut shell liquid (CNSL) feeding on the methane (CH_4_) emission and the ruminal microbiome of Lai Sind beef cattle were investigated. Changes in the methane production and rumen microbiome by CNSL feeding were monitored by a respiration chamber and 16S rRNA gene amplicon sequencing respectively. The results demonstrated that CNSL feeding mitigated 20.2%–23.4% of the CH_4_ emission *in vivo* without apparent adverse effects on feed intake and feed digestibility. The rumen fluid analysis revealed a significant increase in the proportion of propionate in the total short‐chain fatty acids. The relative abundance of methanogen (order *Methanobacteriales*) decreased significantly, indicating the direct inhibitory effect of CNSL on methanogens. The predicted function of the rumen microbiome indicated that carbohydrate and lipid metabolisms including propionate production were upregulated by CNSL feeding, whereas CH_4_ metabolism was downregulated. A network analysis revealed that methanogen changed its partner bacteria after CNSL feeding. The δ^13^C of CH_4_ ranged from −74.2‰ to −66.6‰ with significant fluctuation by CNSL feeding, in agreement with the shift of the rumen microbiome. Our findings demonstrate that CNSL feeding can mitigate the CH_4_ emission from local cattle production systems in South‐East Asia by modifying the rumen microbiome and its function.

## Graphical abstract

CNSL feeding reduced enteric CH_4_ emission from Vietnamese cattle by 20%. CNSL feeding reduced the relative abundance of the major methanogens in the rumen. CNSL feeding also changed the whole rumen microbiome and its function, and the methanogen switched its bacterial partners by CNSL feeding.

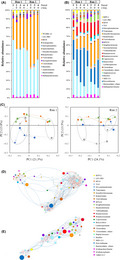

## Introduction

The livestock sector is a significant source of greenhouse gases (GHG), as it produces 5.6–7.5 GtCO_2_e year^−1^, which accounts for approx. 15% of the total anthropogenic GHG emission; the largest source of emission is enteric methane (CH_4_) (1.6–2.7 GtCO_2_e year^−1^), followed by nitrous oxide (N_2_O) emission associated with feed production (1.3–2.0 GtCO_2_e year^−1^) (Herrero *et al*., 2016). To achieve the goals of the Paris Agreement and to tackle global warming, the enteric CH_4_ emission from cattle is one of the key targets for GHG emission mitigation. The demand for meat is currently increasing worldwide. In developing countries that have recently experienced economic growth and changes in consumer preferences, the demand for meat is predicted to double by 2050 (Smith *et al*., 2018). Therefore, to minimize the increase of CH_4_ emissions by the larger cattle population, options for significantly mitigating enteric CH_4_ emissions are urgently needed.

One of the approaches for mitigating enteric CH_4_ emissions is to manipulate livestock diets with various additives. Extensive efforts have already been made in this direction, including studies using chemical compounds that specifically inhibit methanogens (Tomkins *et al*., 2009; Hristov *et al*., 2015), antibiotics (Grainger *et al*., 2008) or electron acceptors (Leng, 2008). Alternatives are natural compounds or agricultural by‐products, which are preferable from the viewpoint of environmental impact and circular agricultural production systems.

Several research groups have reported that the use of cashew nut shell liquid (CNSL) as a feed additive can mitigate enteric CH_4_ emissions by 18%–73% *in vitro* (Watanabe *et al*., 2010; Watanabe *et al*., 2010; Danielsson *et al*., 2014) and by 19.3%–38.3% *in vivo* (Shinkai *et al*., 2012; Konda *et al*., 2019). CNSL is a by‐product of cashew processing that consists mainly of anacardic acid, cardol and some other phenols; it is usually used for brake linings or surface coatings, or more recently for green diesel production (Lomonaco *et al*., 2013; Scaldaferri and Pasa, 2019), and it has an antimicrobial activity for several microbial species (Muroi and Kubo, 1993; Muroi and Kubo, 1996; Green *et al*., 2007; Rivero‐Cruz *et al*., 2011; da Silva *et al*., 2016).

The global cashew nut production in 2017 was 3.97 million t, with 50.6% of the total being produced in Asia, where Vietnam (21.7%) and India (18.8%) being the two largest producers on the Asian continent (FAOSTAT). The effective use of locally produced CNSL within local livestock farming is highly preferable from the point of view of sustainable food production.

Here, we evaluated the effects of CNSL on the CH_4_ emission by Lai Sind, a widely used breed of Vietnamese beef cattle, by a head‐cage respiration chamber technique. We also assessed feed digestibility to understand the effects of CNSL feeding on animal productivity. To determine the effects of CNSL feeding on the organic matter degradation in the rumen and the underlying mechanism, we measured the δ^13^C value of the produced CH_4_ and investigated the rumen microbiome by performing amplicon sequencing of the 16S rRNA gene.

## Results

### Methane emission, ruminal pH, SCFA concentrations and feed digestibility

**Fig. 1:**
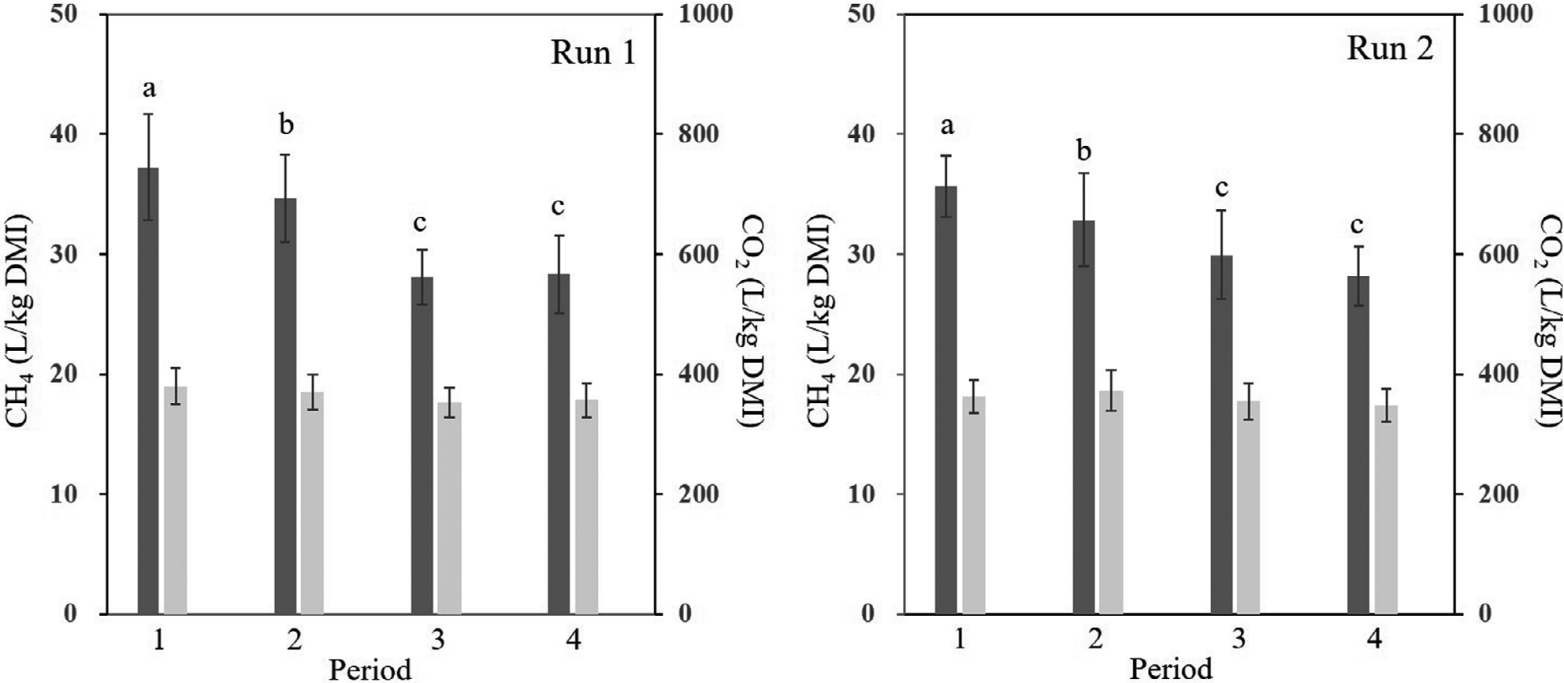
Enteric CH_4_ (*dark grey*) and CO_2_ (*light grey*) emissions per kg dry matter intake (DMI) from Lai Sind cattle with (periods 2–4) and without (period 1) CNSL feeding (*n* = 4). Error bars: standard deviation (SD). Different letters indicate significant differences (*P* < 0.05). Different doses of CNSL were set in Run 1 (4 g/100 kg BW) and Run 2 (6 g/100 kg BW).

The average CH_4_ emissions determined by the head‐cage chamber technique decreased significantly (*P* < 0.05) from 37.2 ± 4.4 l kg^−1^ dry matter intake (DMI) (in period 1 without CNSL feeding) to 28.1 ± 2.3 l kg^−1^ DMI in period 3 (24.5% reduction) and 28.3 ± 3.3 l kg^−1^ DMI in period 4 (23.9% reduction) with CNSL feeding (4 g/100 kg BW) in Run 1. This trend continued in Run 2 with the higher CNSL dose at 6 g/100 kg BW, with average CH_4_ emissions decreasing significantly (*P* < 0.05) from 35.6 ± 2.6 l kg^−1^ DMI (in period 1 without CNSL feeding) to 29.9 ± 3.7 l kg^−1^ DMI in period 3 (16.0% reduction) and 28.2 ± 2.5 l kg^−1^ DMI in period 4 (20.8% reduction; Fig. 1).

The average CO_2_ emission was consistent in both experiments: it was not significantly affected by CNSL feeding. It ranged from 352.7 ± 25.1 l kg^−1^ DMI (period 3) to 379.9 ± 29.5 l kg^−1^ DMI (period 1) in Run 1 and from 347.9 ± 27.2 l kg^−1^ DMI (period 4) to 372.7 ± 34.2 l kg^−1^ DMI (period 2) in Run 2. The DMI was not significantly affected by CNSL feeding; it ranged from 4.06 ± 0.37 (period 1) to 4.25 ± 0.44 kg day^−1^ (period 4) in Run 1 and from 5.61 ± 0.58 (period 2) to 5.90 ± 0.55 kg day^−1^ (period 1) in Run 2.

**Fig. 2:**
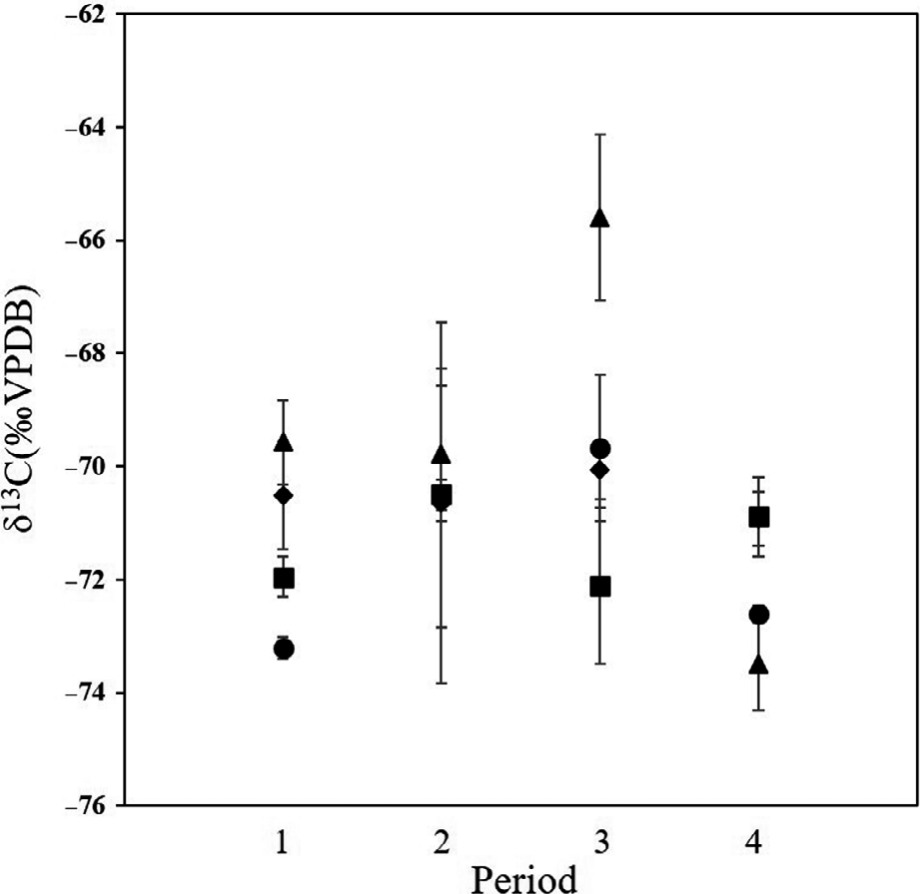
Changes in the δ^13^C values of enteric CH_4_ (Run 1). The δ^13^C values are expressed as relative to the VPDB (Vienna Pee Dee Belemnite). Each symbol (*circle*, *triangle*, *square* and *diamond*) indicates individual cattle. Error bar: SD (*n* = 3).

The δ^13^C values of CH_4_ ranged from −74.2‰ to −66.6‰. The average δ^13^C values of CH_4_ in periods 1, 2, 3 and 4 were −71.1 ± 1.5‰ (*n* = 4, 1σ), −70.4 ± 1.8‰ (*n* = 4, 1σ), −69.7 ± 2.5‰ (*n* = 4, 1σ) and −72.0 ± 1.3‰ (*n* = 4, 1σ) respectively (Fig. 2). The δ^13^C value increased from period 1 to period 3 and decreased from period 3 to period 4. In contrast, the CH_4_ emission decreased from period 1 to period 3 and was almost unchanged from period 3 to period 4 (Fig. 1, Table 1). The variations of the δ^13^C value within individual cattle were higher in periods 2 and 3 compared with periods 1 and 4.

**Table 1:** Nutrient digestibility of four Lai Sind cattle with and without cashew nut shell liquid (CNSL) feeding.

Run	1						*P*	2						*P*
Period	1	SD	3	SD	4	SD		1	SD	3	SD	4	SD	
DM	69.9	4.9	70.6	1.8	71.8	3.6	0.69	64.5	0.6	62.3	2.7	63.4	3.4	0.40
OM	72.9	4.1	73.5	1.6	74.4	3.6	0.73	67.2	0.3	65.8	1.2	67.2	3.0	0.39
CP	78.8	2.3	78.9	3.8	78.4	2.8	0.83	67.9^a^	0.9	66.7^ab^	1.6	64.3^b^	3.2	0.02
EE	80.4	5.3	82.3	2.3	80.5	2.2	0.47	73.4	18.1	55.8	10.8	57.2	11.8	0.16
NDF	70.6	5.4	72.3	1.4	72.5	2.6	0.77	67.1	1.1	65.4	2.7	66.1	2.9	0.48
GE	69.9	4.3	71.4	2.0	71.8	3.3	0.76	64.1	0.5	63.8	1.7	62.6	2.8	0.47

The data are percentages. Different amounts of CNSL were fed in Run 1 (4 g/100 kg BW) and Run 2 (6 g/100 kg BW). Feed without CNSL was fed to cattle as control period 1, and CNSL was fed to the cattle for periods 3 and 4. The detailed experimental schedule can be found in Figure S1. Different superscript letters indicate that the means are significantly different between the periods (*P* < 0.05). CP, crude protein; DM, dry matter; EE, ether extract; GE, gross energy; NDF, neutral detergent fibre; OM, organic matter.

The changes in the rumen parameters (pH, total short‐chain fatty acids [SCFA] and ammonia‐N concentration) are illustrated in Figure S2. The rumen pH remained at a consistent level (6.9 ± 0.1) throughout Run 1, but it increased from 6.9 ± 0.1 to 7.3 ± 0.1 in Run 2. The total SCFA concentration remained at a mostly consistent level that ranged from 52.3 ± 0.5 mM to 68.6 ± 6.4 mM in Run 1, and it also remained at a consistent level in Run 2, but with the much higher variation of 40.5 ± 11.4 mM to 55.9 ± 23.8 mM among the four cattle in both runs; despite the mostly consistent levels of SCFA, small peaks were detected after CNSL feeding (Fig. S3).

We also determined the digestibility of the feeds, and we did not observe any significant effect of CNSL feeding on the dry matter (DM), organic matter (OM), crude protein (CP), ether extract (EE), neutral detergent fibre (NDF) and gross energy (GE) digestibility in Run 1, whereas a significant reduction in CP digestibility was observed in Run 2 (Table 1).

### Diversity and composition of the rumen microbiome

After the quality filtering by a standard DADA2 procedure, 118 783 ± 25 839 reads were obtained for each sample. We evaluated the effects of the CNSL feeding on the rumen microbiome diversity by using six diversity indexes (Brillouin, Chao1, Evenness, Faith's PD, Shannon and Simpson) (Table 2). All six indexes decreased after CNSL feeding. Two indexes showed a significant decrease (*P* < 0.05) in Run 1 with the lower dose (Chao1: from 800.6 to 588.0; Faith's PD: from 53.03 to 45.51), and five indexes showed a significant decrease (*P* < 0.05) in Run 2 with the higher dose (Brillouin: from 5.22 to 4.72; Chao1: from 778.2 to 612.3; Evenness: from 0.787 to 0.738; Shannon: from 7.56 to 6.83; and Simpson: from 0.984 to 0.963), indicating that the CNSL feeding significantly reduced the rumen microbiome diversity.

**Table 2:** Effect of CNSL feeding on the rumen microbiome diversity.

	Run 1				Run 2			
	1	2	3	4	1	2	3	4
Brillouin	4.67	4.65	4.57	4.20	5.22^ab^	5.35^a^	5.04^b^	4.72^c^
Chao1	800.6^a^	741.3^ab^	663.0^ab^	588.0^b^	778.2^a^	760.1^ab^	635.8^ab^	612.3^b^
Evenness	0.700	0.705	0.704	0.661	0.787^a^	0.810^a^	0.785^a^	0.738^b^
Faith's PD	53.03^a^	51.3^ab^	48.27^ab^	45.51^b^	46.34	45.18	42.79	41.88
Shannon	6.75	6.72	6.61	6.08	7.56^ab^	7.74^a^	7.30^b^	6.83^c^
Simpson	0.952	0.961	0.941	0.912	0.984^a^	0.989^a^	0.984^a^	0.963^b^

Different amounts of CNSL were fed in Run 1 (4 g/100 kg BW) and Run 2 (6 g/100 kg BW). Feed without CNSL was fed to cattle as the control at period 1, and CNSL was fed to the cattle for periods 2 through 4. The detailed experimental schedule is presented in Figure S1. Differing superscript letters indicate that the means are significantly different between the periods (*P* < 0.05).

**Fig. 3:**
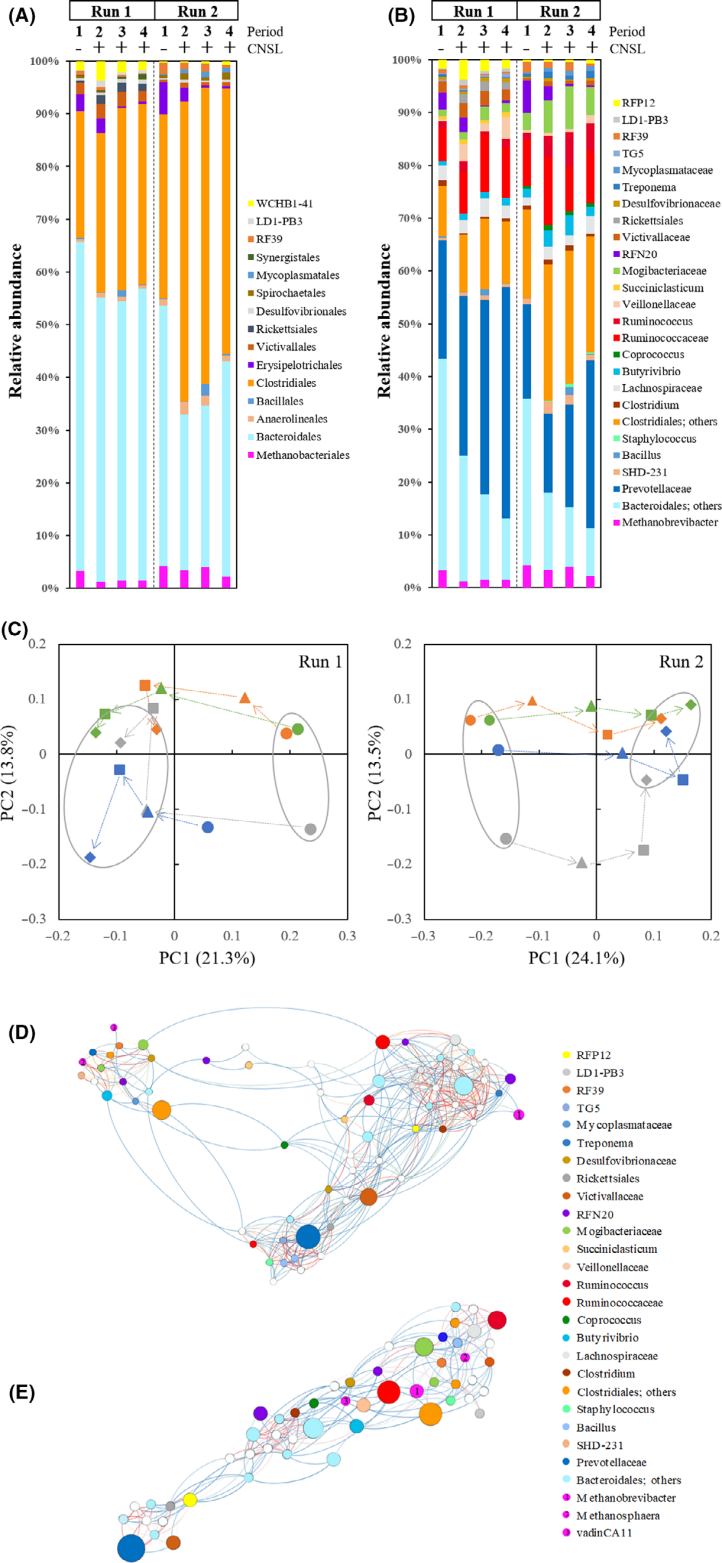
Changes in the ruminal microbiome over time. The ruminal total bacterial/archaeal composition is shown with the order level (A) and family/genus level (B) by the amplicon sequencing of partial 16S rRNA gene. ‘Period’ and ‘CNSL’ indicate the sampling period (after CNSL feeding; period 1 indicates just before CNSL feeding) of ruminal fluid with (+) or without (−) CNSL feeding. (C) Principal component analysis of the ruminal microbiome over time. Each colour indicates a different individual. *Circle:* period 1, *triangle:* period 2, *square:* period 3, *diamond:* period 4. (D, E) Network analysis of the rumen microbiome in periods 1 (D) and 4 (E). Data from both runs were analysed together. The relative abundance of each group is reflected in the size of the circles. Colours of the lines between each circle indicate the correlation coefficient, from low (*red*) to high (*blue*) correlation. Only significant correlations (*r* > 0.5) are shown.

The compositions of the rumen microbiome in the two runs are illustrated in Figure 3. The bar plots with order levels show that the CNSL feeding had a significant impact on the rumen microbiome (Fig. 3A). In the comparison between periods 1 and 4, the relative abundance of the following orders decreased in both runs: *Erysipelotrichales* (3.5 ± 2.1% to 0.6 ± 0.4% in Run 1; 6.2 ± 3.9% to 0.6 ± 0.3% in Run 2), *Bacteroidales* (58.4 ± 5.2% to 51.5 ± 9.5% in Run 1; 47.2 ± 5.8% to 37.5 ± 5.7% in Run 2) and *Methanobacteriales* (3.1 ± 0.9% to 1.4 ± 0.6 in Run 1; 4.2 ± 0.8% to 2.3 ± 0.6% in Run 2). The relative abundance of *Clostridiales* increased (26.2 ± 4.9% to 41.9 ± 11.4% in Run 1; 33.6 ± 2.3% to 46.4 ± 4.1% in Run 2).

Since some major orders such as *Clostridiales* and *Bacteroidales* consist of many important families with different functions, we broke these orders down to the family or genus level and generated another bar plot to observe the effects of the CNSL feeding in more detail (Fig. 3B). The CNSL feeding exerted a consistent effect on the rumen microbiome, although the variation among the individual cattle was high, with CNSL feeding increasing the abundance of the family *Prevotellaceae* (20.8 ± 19.7% to 40.3 ± 13.1% in Run 1; 17.1 ± 3.0% to 29.1 ± 8.0% in Run 2) and decreasing the abundance of other species belonging to *Bacteroidales*. Among the species in the order *Clostridiales*, the relative abundance of the following three families was significantly increased by CNSL feeding: those belonging to *Ruminococcaceae* (7.3 ± 2.0% to 10.4 ± 4.2% in Run 1; 9.6 ± 0.3% to 13.8 ± 1.5% in Run 2), *Veillonellaceae* (0.9 ± 0.3% to 5.0 ± 1.4% in Run 1; 0.4 ± 0.1% to 1.4 ± 0.3% in Run 2) and *Mogibacteriaceae* (1.1 ± 0.3% to 1.5 ± 0.7% in Run 1; 3.0 ± 0.5% to 4.9 ± 1.2% in Run 2). The relative abundance of the following was significantly decreased: the genus *Methanobrevibacter* (3.1 ± 0.9% to 1.4 ± 0.5% in Run 1; 4.0 ± 0.7% to 2.0 ± 0.5% in Run 2), which is the most abundant methanogen, and *RFN20* (3.1 ± 1.9% to 0.4 ± 0.3% in Run 1; 5.9 ± 4.0% to 0.3 ± 0.4% in Run 2), which belongs to the order *Erysipelotrichales*.

The results of the principal component analysis confirmed these clear effects of CNSL feeding (Fig. 3C). The initial rumen microbiomes of the four cattle were located in the same area, and they all fell into another independent area in period 4 samples, which was consistent in both runs. We also conducted a network analysis based on the correlation coefficient between each species for periods 1 and 4 to compare the initial and final rumen microbiome (Fig. 3D). The loss of microbial diversity due to the CNSL feeding significantly changed the shape of the rumen microbiome and made it simpler in period 4. The network analysis data are illustrated based on their correlation and thus the connected species with lines indicating the functional partners that share or hand over the substrates and are functionally related in the micro‐ecosystems.

In period 1, the three detected methanogens fell into two clusters that had high correlations with *Anaeroplasmatales* and *Acholeplasmatales*, whereas in period 4, the methanogens all had high correlations with different species such as *Mogibacteriaceae*, *Ruminococcaceae* and *Christensenellaceae* as their potential functional partners. These results indicate that the CNSL feeding significantly affected the rumen microbiome ecosystem, including the activity of methanogens and their partners. *Prevotellaceae* belonged to different clusters in the two periods, indicating that the induction of propionate production and methanogenic activity are independent of each other.

### Predicted functions and metabolic pathways of the rumen microbiome

**Fig. 4:**
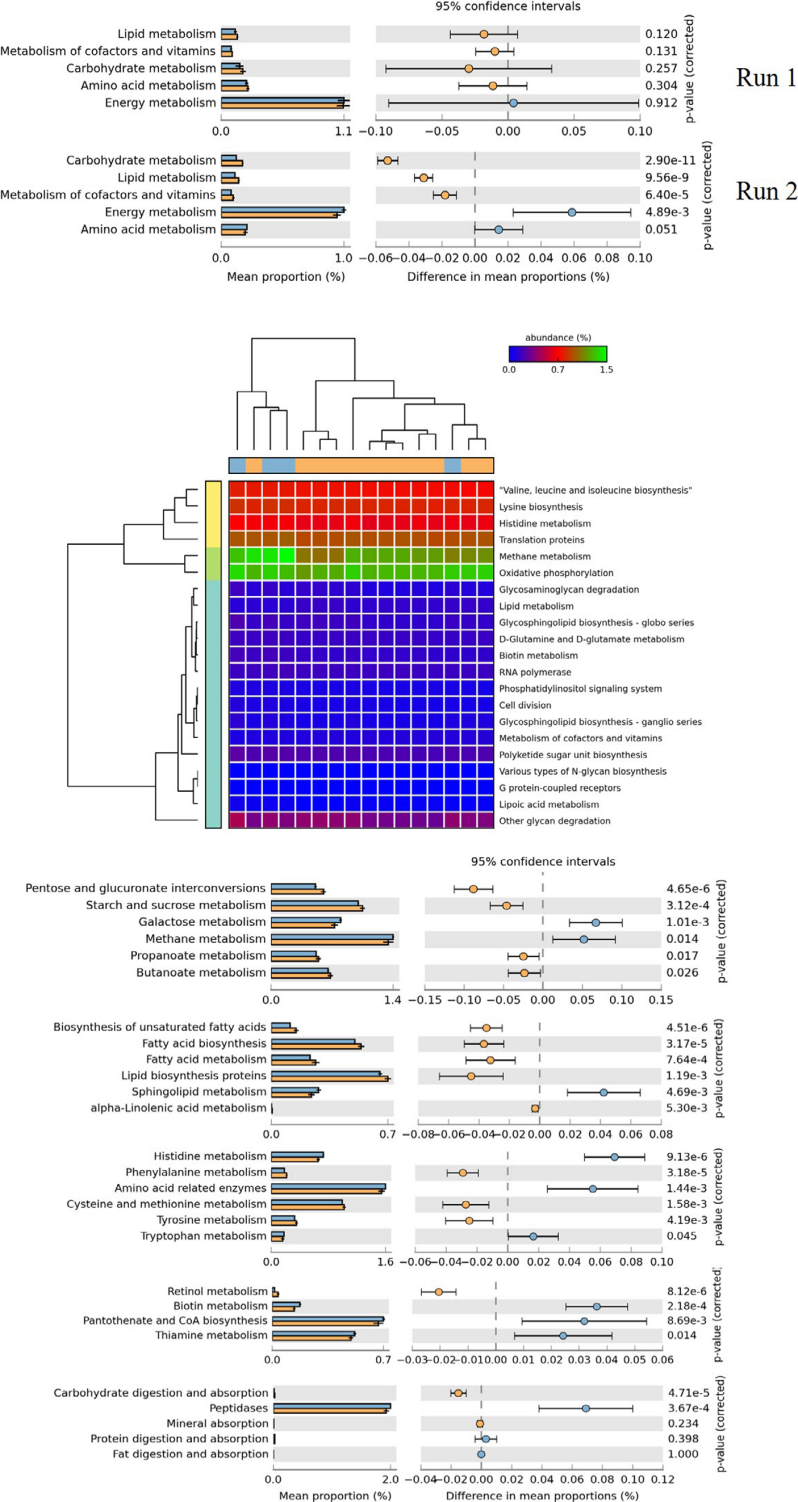
Predicted functions of the ruminal microbiome by PICRUSt. A. The effect of CNSL feeding on ruminal functions related to feed digestion. Only higher hierarchies in KEGG pathways were selected. B. Significantly changed features by CNSL feeding in Run 1. *Orange* and *light blue* at the bottom of the dendrogram indicate the results with (periods 2–4) and without (period 1) CNSL feeding. C. Significantly changed features by CNSL feeding in Run 2. The lower hierarchy in KEGG pathways that was related to feed digestion and nutrient intake was selected.

To compare the functional potential of the rumen microbiome with and without CNSL feeding, we used PICRUSt to predict the function of the rumen microbiome (Fig. 4). The CNSL feeding significantly (*P* < 0.05) affected 34 features in Run 1 and 92 of the 328 features in total. The two runs showed similar trends, with CNSL feeding increasing the lipid, carbohydrate and vitamin metabolisms. However, these differences became significant (*P* < 0.01) only in Run 2 at the higher CNSL dose (Fig. 4A). In addition, the samples with and without CNSL feeding were well separated in the dendrogram (Fig. 4B), indicating that the predicted functions in the rumen microbiome with and without CNSL feeding were significantly different. The active features affected by the CNSL feeding included the CH_4_ metabolism (Run 1, *P* = 0.045; Run 2, *P* = 0.014). Thus, the CNSL feeding clearly affected the CH_4_ production and related metabolism by the rumen microbiome, which agrees well with the reduced CH_4_ production after CNSL feeding (Fig. 1).

Other metabolisms related to feed degradation and important metabolisms for cattle nutrients are summarized in Figure 4C. This prediction indicates that CNSL feeding might activate the metabolism of starches (*P* < 0.01), including cellulose, and the metabolism and biosynthesis of fatty acids, including propionate or butyrate (*P* < 0.05). In regard to the metabolism of amino acids, the effect of the CNSL feeding differed among the amino acid species. Histidine (*P* < 0.01) and tryptophan (*P* < 0.05) metabolism appeared to be suppressed by the CNSL feeding, whereas the phenylalanine, cysteine, methionine and tyrosine metabolisms were activated (*P* < 0.01).

## Discussion

The world cattle population increased by 13.4% over the last 20 years, from 1.32 billion in 1998 to 1.49 billion in 2018 (FAOSTAT). In South‐East Asia, the rate of increase within the same time frame was much higher at 37.5%. Greater numbers of cattle can lead to greater environmental pollution, including GHG emissions, and thus, there is an urgent need for a feasible GHG mitigation strategy for local production systems. We focused herein on the GHG mitigation potential of the locally available feed resource CNSL, and our results demonstrated that the feeding of CNSL to Lai Sind, a breed of Vietnamese local cattle, successfully reduced the CH_4_ emission per kg DMI by 20.8%–23.9% (Fig. 1).

We fed the cattle CNSL at two different doses, but the reduction rate was higher at the lower dose, indicating that this level of ~ 20% is the maximum mitigation potential. Since we had a sufficiently long buffer time with 505 days between runs, we suspect that there was a minimum carryover effect for Run 2. This reduction rate is comparable to the rate of 19.3% determined by respiration chamber measurements in an *in vivo* study of Holstein dairy cattle (Shinkai *et al*., 2012) with the same CNSL dose level. More recently, a similar in vitro experiment using incubation of rumen fluid from Thai native cattle and swamp buffalo revealed a CH_4_ mitigation potential of 53%–73% (Konda *et al*., 2019). Although it is in vitro study that did not use a respiration chamber and therefore a direct comparison with our study is not possible, these previous results and our present findings indicate that CNSL can be effective regardless of the genetic background of the cattle.

The reduction rate in the present study was lower than that in studies using the chemical inhibitor 3NOP (25%–32%; Hristov *et al*., 2015) or nitrate (11.8%–29.4%; Newbold *et al*., 2014) but significantly higher than that in a study using monensin (7–9%; Odongo *et al*., 2007), which is widely used in the beef production system in the United States, and also significantly higher than those in studies using other natural resources such as tannin (0%–11%; Beauchemin *et al*., 2007a; Bhatta *et al*., 2013) or lipid sources (17%; Beauchemin *et al*., 2007b). More recently, some seaweed species were shown to be effective to mitigate ruminal CH_4_ emission (26.4%–67.1%), but the feed intake was also significantly reduced (10.8%–38.0%), which may reduce the animals' productivity (Maia *et al*., 2016; Kinley *et al*., 2016; Roque *et al*., 2019). Vietnam is the world's largest producer of cashew, and the production of this crop has increased in recent years, with a cultivation area of 283 thousand ha and production at 2.6 million t in 2018 (FAOSTAT). The cashew shell as a by‐product is abundantly available with low cost for transportation, which is promising for its use as a feed additive in the 5.8 million cattle in Vietnam.

The results of our CH_4_ isotopic analysis indicated that the CNSL feeding also affected the δ^13^C values of the emitted CH_4_ (Fig. 2). The range of the δ^13^C values (−74.2‰ to −66.6‰) fell within the range of earlier investigations (Rust, 1981; Levin *et al*., 1993; Bilek *et al*., 2001; Klevenhusen *et al*., 2009, 2011), indicating that the main methanogenic pathway is CO_2_ reduction (Whiticar, 1999). This agrees well with the results of our microbiome analysis, which demonstrated that the major part of the ruminal methanogens was *Methanobrevibacter* (Fig. 3), since it is known as a hydrogenotrophic methanogen (Leahy *et al*., 2010). Temporary high variations and enriched δ^13^C values were observed after the CNSL feeding (periods 2 and 3), which was the opposite of the result in a study of methanogenesis inhibition by 3‐NOP (Lopes *et al*., 2016). Although we did not measure the δ^13^C of precursor CO_2_, these results indicate that the CNSL feeding affected the feed digestion and its isotope fractionation from feed to CO_2_ and further to CH_4_ in some individual cattle. This might have been due to the significant disturbance of the rumen microbiome (Fig. 3) by the CNSL feeding, but this is only circumstantial evidence, and much more detailed measurements (including those of precursors) are required in future studies.

We observed that the effects of the CNSL feeding on the ruminal total SCFA, ammonia concentration and pH were small. The pH tended to increase slightly only in Run 2, whereas the total SCFA concentration tended to decrease in both runs with very high variation among the individual cattle (Fig. S2). Moreover, the CNSL feeding affected the proportion of SCFA, and it significantly increased the proportion of propionate (Fig. S3), which agrees with other reports of CNSL feeding experiments (Shinkai *et al*., 2012; Konda *et al*., 2019). The effect of the CNSL feeding on feed digestibility was also small. We measured the DM, OM, CP, EE, NDF and GE and observed no significant effect on any of them in Run 1 with the lower dose, indicating that CNSL feeding does not have a negative effect on feed digestion in the rumen. Since this experiment was performed only for short periods (i.e. 48 days for each run), another experiment with longer periods is needed to determine whether this effect can be sustained.

In Run 2 with the 50% higher dose, the digestibility of CP was significantly reduced (Table 1). Moreover, although the difference was not significant, the digestibility of EE in the CNSL‐fed period (periods 3 and 4) also tended to be lower than that of the CNSL‐free period 1. Since the CH_4_ mitigation potential was in the same range, the lower dose in Run 1 is preferable for practical use. These results indicate that CNSL feeding can modify the rumen fermentation characteristics and change the SCFA profiles without adversely affecting the feed digestion efficiency under the optimum dose, which is very important from the point of view of animal production.

The whole‐genome sequence information of ruminal bacteria has been substantially improved, enabling functional estimations by using the next‐generation sequencing data of marker genes (Langille *et al*., 2013; Seshadri *et al*., 2018). High‐throughput sequencing of partial bacterial/archaeal 16S rRNA genes reveals the effects of CNSL feeding on the composition of the rumen microbiome and the metabolic functions in detail. Here, the CNSL feeding clearly reduced the relative abundance of the dominant methanogen *Methanobrevibacter*, which directly explains the 20% reduction in CH_4_ emission. The bacterial community in the Lai Sind cattle rumen is dominated by two orders, *Bacteroidales* and *Clostridiales*, which together account for > 80% of the whole sequences. CNSL feeding affected the balance of two dominant orders, increasing the *Clostridiales* abundance and decreasing the *Bacteroidales* order (Fig. 3A).

More specifically, while the abundance of the order *Bacteroidales* was decreased by CNSL feeding, the relative abundance of *Prevotellaceae*, the major group within *Bacteroidales*, was significantly increased (Fig. 3B). This group is known to be able to produce propionate through fumarate reduction and succinate production pathway (Louis and Flint, 2017), which is a pathway that requires vitamin B12 and consumes hydrogen (Strobel, 1992; Ungerfeld, 2013). Our present analyses revealed that the CNSL feeding also significantly increased the abundance of *Ruminococcaceae*; some species belonging to this family produce succinate as their end product (Macfarlane and Gibson, 1997). The abundance of another minor group, *Veillonellaceae*, which is also known to produce propionate through the succinate pathway (Reichardt *et al*., 2014), was also significantly increased after the CNSL feeding.

The predicted functions of the rumen microbiome also suggest that the CH_4_ metabolism was significantly downregulated and the propionate or butyrate metabolism was upregulated by the CNSL feeding (Fig. 4B and C). Although the proportion of butyrate in the total SCFA was not changed in our measurements (Fig. S2), all of these results well underpin the concept that with the feeding of CNSL, the cattle's CH_4_ emission was reduced, while the propionate proportion in total SCFA was increased. We propose the following mechanism to explain these results.

First, the CNSL feeding directly inhibited the methanogenesis by the major methanogen *Methanobrevibacter* and reduced the relative abundance and activity of this species. Second, the inhibition of methanogenesis led to a greater excess of hydrogen in the rumen (not measured in this study), since methanogenesis is a hydrogen‐consuming process that uses CO_2_ as an electron acceptor (Lyu *et al*., 2018). Third, some of the excess hydrogen that was not consumed by methanogenesis was consumed by an alternative hydrogen sink – namely the production of propionate through the succinate pathway (or the biohydrogenation of unsaturated fatty acids) – and the bacterial species that have enzymes that are required for these reactions proliferated in the rumen ecosystem.

The CNSL feeding also affected other bacterial species, increasing the relative abundance of *Butyrivibrio*. One of the functions of *Butyrivibrio* in the rumen is the biohydrogenation of unsaturated fatty acids (Maia *et al*., 2010; Dewanckele *et al*., 2018). Although it is estimated to be a minor pathway, biohydrogenation can be an alternative hydrogen sink, which might be activated in the methanogenesis‐inhibited rumen ecosystem by CNSL feeding.

The results of our comparison of the network analysis outputs between periods 1 (without CNSL feeding) and 4 (Fig. 3D) indicate that the CNSL feeding significantly changed the structure of the rumen microbiome, making it simpler, which agrees with the results of the diversity index (Table 2). In both periods, *Prevotella*, which was estimated to be the main propionate producer whose abundance was increased after CNSL feeding, was completely apart from all three methanogens. Although this result was not surprising, this strongly suggests that the methanogenesis and the propionate production by *Prevotella* are functionally independent. *Prevotella* tended to have a close relationship with *Victivallaceae*, *Rickettsiales*, *Acinetobacter* and *TG5* (*Synergistales*) in both periods 1 and 4. Previously, Pybus and Onderdonk (1998) showed that *Prevotella bivia* can provide amino acids to *Peptostreptococcus* species, which cannot grow in pure culture. Based on this, it may be possible that *Prevotella* species have this kind of symbiotic relationship with these groups stated above in the complex rumen ecosystem. These results suggest that the function and partners of *Prevotella* are stable irrespective of CNSL feeding.

In contrast, the most abundant methanogen, *Methanobrevibacter,* changed its partners after the CNSL feeding. In period 1, two of the three bacterial species that had a significant relationship with *Methanobrevibacter* belonged to the orders *Anaeroplasmatales* and *Acholeplasmatales*, both of which belong to the class *Mollicutes*. Although their functions are not well understood, these species can be free‐living and have some potential to be parasites of ruminal fungi or protozoa (Joblin and Naylor, 2002). The reconstructed genome of their close relatives revealed that these two species are fermenters and that they utilize simple sugars and possess hydrogenase (Skennerton *et al*., 2016), indicating that they have some potential to supply hydrogen to the methanogens. The relative abundance of these species was very low (0.1%–0.4%), and their abundance dropped significantly (< 0.1%) after CNSL feeding.

In period 4, species with a significant correlation with Methanobrevibacter changed to other groups such as *Mogibacteriaceae*, *Ruminococcaceae* and *Christensenellaceae* (Fig. 3D). They also had a close relationship with the minor methanogens *Methanosphaera* and *Methanomassillicoccaceae* in period 1, indicating that the CNSL feeding forced *Methanobrevibacter* to switch its partner. These results also suggest that the hydrogen supply from *Mollicutes* species to *Methanobrevibacter* was inhibited and that *Methanobrevibacter* changed and shared its metabolic partners with other methanogens in the CNSL‐fed rumen. This change agrees well with the reduced CH_4_ emission and *Methanobrevibacter* population in the CNSL‐fed rumen with lower diversity.

The prediction of the ruminal microbiome function by PICRUSt showed that CNSL feeding can affect not only important features of feed digestion (such as carbohydrate or lipid metabolisms) but also many fundamental processes of the microbes (such as cell division, oxidative phosphorylation and translation proteins) (Fig. 4B). Among them, it is noteworthy that metabolism of carbohydrate, lipid, cofactors and vitamins tended to be upregulated, especially in Run 2 with the higher CNSL dose (Fig. 4A). In the detailed KEGG pathways, the metabolisms of starch and sucrose (including cellulose degradation) were significantly upregulated by the CNSL feeding, whereas the protein digestion and peptidase tended to be higher in the ruminal microbiome without CNSL (Fig. 4C).

These predictions partly agree with the digestibility of the feed; the CP digestibility in Run 2 was significantly affected by CNSL feeding (Table 1), and these predictions indicate that CNSL feeding has some potential for better feed digestion or utilization by the significantly changed rumen microbiome. In addition, the prediction results regarding the ruminal microbiome function biosynthesis of fatty acids (including unsaturated fatty acids) and their metabolism were also significantly upregulated (Fig. 4C), in contrast to the trend for total SCFA (Fig. S2), which had high variation but tended to be decreased by CNSL feeding. Another study also showed that the total SCFA was sometimes significantly lower than in the control (Shinkai *et al*., 2012). Since the SCFA produced in the rumen can be absorbed from the rumen, these opposite results should be further examined with future experiments.

In conclusion, the feeding of CNSL successfully reduced the enteric CH_4_ emission from the Vietnamese cattle breed Lai Sind with high reproducibility. The hydrogen consumption in the rumen was switched from methanogenesis to other pathways such as the propionate production pathway or other minor hydrogen sinks. The feed digestion efficiency was not affected by the CNSL feeding, but the prediction of ruminal microbiome function indicated that some microbial pathways for fibre or lipid metabolisms might be upregulated. Since CNSL is abundant in the local agricultural production system in Vietnam, these results pave the way for the efficient utilization of cashew nutshells as a by‐product of the world's primary cashew industry. This approach can contribute to the mitigation of the enteric CH_4_ emissions in South‐East Asian countries.

## Experimental procedures

### Animals, diet composition, CNSL feeding and CH_4_ emission measurement

A CNSL feeding experiment was performed twice on the experimental farm at Can Tho University (CTU). Four male Lai Sind cattle with the mean body weights of 246.1 ± 22.6 kg (Run 1, low dose) and 375 ± 36 kg (Run 2, high dose) were used for the CNSL feeding experiments with 48 days (14 days for adaptation, 7 days of feeding without CNSL and 27 days of feeding with CNSL) for each run (Fig. S1). There was a buffer time (505 days) between the runs to minimize the carryover effect of CNSL feeding. During the experimental period, the cattle were kept individually in their stalls and fed a typical local feed based on rice straw and concentrate (60:40). The cattle were fed 1.7% of dry matter (DM) per kg body weight per day. Details of the feeding composition are summarized in Table S1.

The cattle were fed at 09:00 and 17:00 each day and provided with clean drinking water. Cashew nut shell liquid (CNSL) at the low dose of 4 g/100 kg body weight (BW) for Run 1 and at the high dose of 6 g/100 kg BW for Run 2 was provided by Idemitsu Kosan (Japan) as a mixture with silica powder and was mixed with molasses and concentrate before feeding. Only silica powder was fed during the control period. The cattle were cared for in accordance with institutional guidelines and the Guidelines for Proper Conduct of Animal Experiments (Science Council of Japan, June 1, 2006).

The emission of CH_4_ was measured by the ventilated hood system installed at the CTU experimental farm (Sakai *et al*., 2016). Briefly, blowers delivered a constant airflow from the hood into the measurement system, and the concentrations of CO_2_ and CH_4_ were measured continuously by a non‐dispersive infrared sensor (IR‐200; Yokogawa Electric, Tokyo, Japan). The detailed schedule of the experiment is presented in Figure S1.

Briefly, experimental feed was fed to all four cattle for 3 weeks for adaptation. Four CH_4_ measurement periods consisting of 5 days each were used: periods 1 to 4. The cattle were always placed in the chamber system for the entire days during each period. The cattle were fed the control feed (without CNSL) only during Period 1, and throughout periods 2 to 4, they were fed CNSL‐containing feed. During the experimental period, feed intake was recorded based on the given amount of feed after correction of leftovers, and rumen fluid (collected after the morning feeding, around 10 am), total faeces and urine were collected, weighed and stored in a freezer (−20°C) for further analysis.

### Stable isotopic analysis of enteric CH_4_

Gas samples for isotopic signature measurement were taken 2 h after the morning feeding. Each period had three sampling times (Fig. S1). The stable carbon isotope ratio of CH_4_ was measured by gas chromatography combustion isotope ratio mass spectrometry (GC‐C‐IRMS). Methane in the samples was separated from interfering components (CO) using a column packed with a 5A molecular sieve (10 mm ID × 500 mm length, 30/60 mesh; GL Sciences, Tokyo) before it was concentrated in a cryofocusing trap. The δ^13^C value was calculated as shown below:

(1) $$\delta^{13}C=\left({\left({}^{13}C/{}^{12}C\right)}_{\text{sample}}/{\left({}^{13}C/{}^{12}C\right)}_{\text{VPDB}‐1}\right)\times 1000$$

Here, VPDB stands for Vienna Pee Dee Belemnite, the international standard for the ^13^C/^12^C ratio. We used 1000 ppm CH_4_ in He (Taiyo Nissan, Japan) with δ^13^C = −39.56% as the working standard (Yamada *et al*., 2005). The precision of the δ^13^C value of CH_4_ was estimated as < 0.4% (*n* = 10, 1σ) based on repeated analyses of the standards. The differences between the measured concentrations and the δ^13^C of duplicate air samples were 0.1%–0.4%.

### Chemical analyses of the feed, faeces, urine and rumen fluid samples

For the analyses of the feed, faeces and urine, the dry matter was measured after drying the samples at 105°C for 24 h. The levels of crude protein (CP), ether extract (EE) and crude ash were determined according to the standard method (Chemists and Horwitz, 1990). The gross energy was determined by an automatic adiabatic bomb calorimeter (C6000; IKA‐Werke, Staufen, Germany). The neutral detergent fibre exclusive of residual ash without inclusion of sodium sulfite (NDF) was determined according to the method of (Van Soest *et al*., 1991). The apparent digestibilities for nutrients were calculated according to the formula: (nutrient consumed − nutrient in faeces)/nutrient consumed.

Rumen fluid samples were taken orally by using the stomach tube. Ruminal pH was measured with a calibrated electrode (Horiba, Fukuoka, Japan). The ammonium concentration of the rumen fluid was determined by a standard colorimetric method using a spectrophotometer (SmartSpec Plus; Bio‐Rad, Hercules, CA) (O'dell, 1993). The short‐chain fatty acids (SCFAs) of the rumen fluid were measured by high‐performance liquid chromatography (Ultimate 3000; Thermo Fisher Scientific, Waltham, MA) (Guerrant *et al*., 1982).

### DNA extraction and 16S rRNA gene amplicon sequencing

DNA was extracted from 2 ml of rumen fluid samples using Isofecal for Beads Beating (Nippon Gene, Tokyo), quantified by NanoDrop Lite (Thermo Fisher) and stored at −20°C until further analysis.

Partial fragments of the 16S rRNA gene (the V4 hypervariable region) were amplified by two‐step polymerase chain reaction (PCR). Primers 515F and 806R (Caporaso *et al*., 2011) with Illumina adapter overhang sequences were used for the first‐round PCR with 20 cycles, and indexes were attached to the amplicon with eight additional cycles. Each 20‐µl PCR mixture contained 0.2 µl TaKaRa Ex Taq HS DNA polymerase (TaKaRa Bio, Shiga, Japan) with 2 µl of buffer (10 × buffer), 1.6 µl of 2.5 mM dNTP mix, 1 µl of each forward and reverse primer (10 mM), and 1 µl of template DNA.

The first‐round PCR conditions were as follows: 94°C for 2 min; 20 cycles of 94°C for 30 s, 50°C for 30 s and 72°C for 30 s; and a final round of 72°C for 5 min. The PCR products were purified using an Agencourt AMPure XP purification system (Beckman Coulter, Indianapolis, IN) and used for the second‐round PCR with the following conditions: 94°C for 2 min; 8 cycles of 94°C for 30 s, 60°C for 30 s and 72°C for 30 s; and a final round of 72°C for 5 min. Tag‐indexed PCR products were purified again, and their quality and quantity were checked by an Agilent 2100 Bioanalyzer (Agilent, Santa Clara, CA) and a Qubit 2.0 Fluorometer and dsDNA HS Assay Kit (Life Technologies, Carlsbad, CA) respectively. Qualified amplicons were pooled in equal amounts and sequenced with a 250‐bp paired‐end sequencing protocol (Illumina, San Diego, CA).

Raw sequence reads were processed by qiime 2‐2019.7 (Bolyen *et al*., 2019). Paired‐end sequences were merged and quality‐filtered by dada2 (Callahan *et al*., 2016), and the denoised feature table and amplicon sequence variants (ASVs) were used for the taxonomic diversity analysis. Taxonomic classifications were assigned using a naïve Bayes classifier trained on the Greengenes 13_8_99% database, and mitochondria or chloroplast sequences were removed (Bokulich *et al*., 2018). The statistical analyses for the diversity metrics and the principal component analysis were performed through QIIME 2 (diversity ‘core‐metrics‐phylogenetic’). We used ‘Sparse Cooccurrence Network Investigation for Compositional data’ (SCNIC) in qiime 2 (q2‐SCNIC) to perform the network analysis. The correlation network was built using SparCC; the network was built using edges with a correlation coefficient of at least 0.5, and network was visualized by Gephi.

PICRUSt was used for predicting the function of the rumen microbiome (Langille *et al*., 2013). The closed‐reference OTUs were normalized by copy number, and a new matrix of predicted functional categories was created with the KEGG database. We used STAMP to analyse the PICRUSt output file (Parks *et al*., 2014). The DNA sequences from this study were deposited in the DDBJ Sequence Read Archive (accession numbers: DRR225289 to DRR225230).

### Statistical analyses

All data were analysed by a one‐way analysis of variance (ANOVA) using the general linear model procedure described by SAS (SAS Institute, 2001nstitute, 2001). The statistical model included treatment, cattle and period. Tukey's multiple range comparison test was used to separate the means. A *P*‐value < 0.05 was considered significant.

## Conflicts of interest

None declared.

## Supporting information


**Fig. S1.** Detailed schedule of the feeding experiment. Check marks indicate that sampling or measurements were done. Grey columns indicate the five‐day metabolic tests with CH_4_ emission measurements. Body weights were measured at the beginning and the end of each metabolic test. Methane emission was measured continuously with head cage chambers. Feces and urine samples were analyzed for the digestibility measurements. Rumen fluid samples were used for pH, NH_4_^+^‐N, and SCFA concentration measurements.
**Fig. S2.** Change of the rumen parameters (pH, SCFA and NH_4_^+^‐N concentrations) during the experimental period. Circles indicate total SCFA concentrations, triangles indicate the NH_4_^+^‐N concentrations, and squares indicate the pH. Error bars indicate the standard deviations (*n* = 4). Arrows indicate the starting time point of CNSL feeding.
**Fig. S3.** Change of the composition of total SCFA (in % of total SCFA concentration). Circles indicate the acetate, squares indicate the propionate, and triangles indicate the butyrate. Error bars indicate the standard deviation (*n* = 4). Arrows indicate the starting time point of CNSL feeding.
**Table S1.** Feed composition (Run1).
**Table S2.** Feed composition (Run2).
