# Supplementary material for: Network analysis and functional estimation of the microbiome reveal the effects of cashew nut shell liquid feeding on methanogen behaviour in the rumen
Source: Microb Biotechnol. 2020 Nov 9;14(1):277–90. doi: 10.1111/1751-7915.13702 (PMC7888476; doi:10.1111/1751-7915.13702)
Supplement: Supplementary file 1 — Fig. S1. Detailed schedule of the feeding experiment. Check marks indicate that sampling or measurements were done. Grey columns indicate the five‐day metabolic tests with CH4 emission measurements. Body weights were measured at the beginning and the end of each metabolic test. Methane emission was measured continuously with head cage chambers. Feces and urine samples were analyzed for the digestibility measurements. Rumen fluid samples were used for pH, NH4 +‐N, and SCFA concentration measurements. Fig. S2. Change of the rumen parameters (pH, SCFA and NH4 +‐N concentrations) during the experimental period. Circles indicate total SCFA concentrations, triangles indicate the NH4 +‐N concentrations, and squares indicate the pH. Error bars indicate the standard deviations (n = 4). Arrows indicate the starting time point of CNSL feeding. Fig. S3. Change of the composition of total SCFA (in % of total SCFA concentration). Circles indicate the acetate, squares indicate the propionate, and triangles indicate the butyrate. Error bars indicate the standard deviation (n = 4). Arrows indicate the starting time point of CNSL feeding. Table S1. Feed composition (Run1). Table S2. Feed composition (Run2). [file MBT2-14-277-s001.docx]

**Mitigation of enteric methane emission from Vietnamese beef cattle by cashew nut shell liquid feeding and its effect on the rumen microbiome**

Koki Maeda^1^*, Nguyen Van Thu^2^, Tomoyuki Suzuki^1,3^, Keita Yamada^4^, Kushi Kudo^4,5^, Chie Hikita^6^, Le Van Phong^2^, Nguyen Minh Chon^2^ and Naohiro Yoshida^4,7^

^1^Crop, Livestock & Environment Division, Japan International Research Center for Agricultural Sciences (JIRCAS), 1-1 Ohwashi, Tsukuba, Ibaraki 305-8686, Japan

^2^Faculty of Agriculture, Can Tho University, Campus II, 3/2 St., Ninh Kieu, Can Tho, Vietnam

^3^Central Region Agricultural Research Center, National Agriculture and Food Research Organization (NARO), 768 Senbonmatsu, Nasu-shiobara, Tochigi 329-2793, Japan

^4^Department of Environmental Chemistry and Engineering, Tokyo Institute of Technology, 4259 Nagatsuta, Midori-ku, Yokohama 226-8502, Japan

^5^Faculty of Human Development and Environment, Kobe University, 3-11 Tsurukabuto, Nada-ku, Kobe 657-8501, Japan

^6^Idemitsu Kosan, Co. Ltd, 2-1 Midorigahara, Tsukuba, Ibaraki 300-2646, Japan

^7^Earth-Life Science Institute, Tokyo Institute of Technology, 2-12-1 Ookayama, Meguro-ku, Tokyo 152-8550, Japan

Maeda et al., Fig. S1

Fig. S1: Detailed schedule of the feeding experiment. Check marks indicate that sampling or measurements were done. Grey columns indicate the five-day metabolic tests with CH_4_ emission measurements. Body weights were measured at the beginning and the end of each metabolic test. Methane emission was measured continuously with head cage chambers. Feces and urine samples were analyzed for the digestibility measurements. Rumen fluid samples were used for pH, NH_4_^+^-N, and SCFA concentration measurements.

Maeda et al., Fig. S2

Fig. S2: Change of the rumen parameters (pH, SCFA and NH_4_^+^-N concentrations) during the experimental period. Circles indicate total SCFA concentrations, triangles indicate the NH_4_^+^-N concentrations, and squares indicate the pH. Error bars indicate the standard deviations (n=4). Arrows indicate the starting time point of CNSL feeding.

Maeda et al., Fig. S3

Fig. S3: Change of the composition of total SCFA (in % of total SCFA concentration). Circles indicate the acetate, squares indicate the propionate, and triangles indicate the butyrate. Error bars indicate the standard deviation (n=4). Arrows indicate the starting time point of CNSL feeding.

Maeda et al., Table S1

| Table S1 Feed composition (Run1) | | | | | |
| --- | --- | --- | --- | --- | --- |
|  |  | CP | EE | NDF | TDN |
|  | % DM | % DM | % DM | % DM | % DM |
| Rice straw | 60.0 | 3.0 | 1.0 | 42.8 | 39.6 |
| Concentrate | 40.0 |  |  |  |  |
| Soybean meal | 15.0 | 6.2 | 0.3 | 4.3 | 10.3 |
| Rice bran | 4.0 | 0.4 | 0.3 | 1.3 | 2.5 |
| Broken rice | 16.2 | 1.2 | 0.3 | 1.0 | 11.7 |
| Molases | 4.0 | 0.2 | 0.0 | 0.0 | 2.8 |
| DCP | 0.4 | 0.0 | 0.0 | 0.0 | 0.0 |
| Salt | 0.4 | 0.0 | 0.0 | 0.0 | 0.0 |
| total | 100 | 11.0 | 2.0 | 49.4 | 67.0 |

Maeda et al., Table S2

| Table S2 Feed composition (Run2) | | | | | |
| --- | --- | --- | --- | --- | --- |
|  |  | CP | EE | NDF | TDN |
|  | % DM | % DM | % DM | % DM | % DM |
| Rice straw | 60.0 | 2.9 | 0.9 | 39.1 | 36.5 |
| Concentrate | 40.0 |  |  |  |  |
| Soybean meal | 15.0 | 6.3 | 0.3 | 4.4 | 9.7 |
| Rice bran | 4.0 | 0.4 | 0.4 | 1.2 | 2.8 |
| Broken rice | 16.2 | 1.5 | 0.6 | 1.1 | 11.3 |
| Molases | 4.0 | 0.2 | 0.0 | 0.0 | 2.8 |
| DCP | 0.4 | 0.0 | 0.0 | 0.0 | 0.0 |
| Salt | 0.4 | 0.0 | 0.0 | 0.0 | 0.0 |
| total | 100 | 11.3 | 2.2 | 45.9 | 63.2 |
